# Characterization of the mitochondrial genome of *Eutomostethus vegetus* Konow, 1898 (Hymenoptera: Tenthredinidae) and phylogenetic analysis

**DOI:** 10.1080/23802359.2020.1797563

**Published:** 2020-07-28

**Authors:** Ya Li, Meicai Wei, Jiafen Liu, Gengyun Niu

**Affiliations:** College of Life Sciences, Jiangxi Normal University, Nanchang, China

**Keywords:** Mitochondrial genome, phylogenetic analysis, Tenthredinidae

## Abstract

The nearly complete mitochondrial genome of *Eutomostethus vegetus* Konow, 1898 was high-quality assembled. Several gene rearrangement events were observed in IQM gene cluster by comparing with the inferred insect ancestral mitochondrial genome. Phylogenetic analysis demonstrated the position of *Eutomostethus vegetus* in the Tenthredinidae and showed that Blennocampinae is a sister group to Fenusinae.

*Eutomostethus* Enslin, 1914 is a member of Tomostethini of Blennocampinae (Wei and Nie 1998). The genus is the largest one of Blennocampinae, and about 105 valid species have been described in the world. Species of the genus are the common pest of the plants of Gramineae, including *Poa*, *Juncus*, and *Phyllostachys*, etc. Here, we described the nearly complete mitochondrial genome of *E. vegetus* Konow, 1898, as the first one of Blennocampinae to advance our understanding of its phylogenetic status within Tenthredinidae.

Specimen (CSCS-Hym-MC0184) was deposited in the Asia Sawfly Museum, Nanchang (ASMN). It was collected at Mt. Lu (29.57°N, 115.96°E), Jiangxi province in China, in April 2019. Whole genomic DNA was extracted from the thorax muscle of specimen (SAMN15311680) using the DNeasyR Blood & Tissue Kits (Qiagen, Valencia, CA). Genomic DNA was sequenced by the high-throughput Illumina Hiseq 4000 platform, yielding a total of 48,582,996 raw reads (SRR12057813). One contig was generated by MitoZ (Meng et al. 2019), and then was thoroughly checked by assembly using *Hemibeleses tianmunicus* (unpublished) and *Periclista xanthosomata* (unpublished) as references (coverage were 2,787,422 and 3954, respectively). We used part of *nad2* (100 bp), *trnI* (66 bp), *trnQ* (70 bp) as reference sequences to examine the longest interval region further. By consistently obtaining similar coverage of the assembly contigs, we were able to confirm the 680 bp non-coding region between *trnQ* and *trnI*. Annotations were generated in MITOS web server (Bernt et al. 2013) and revised when necessary. The accession number of BioProject is PRJNA640463.

The nearly complete mitochondrial genome of *E. vegetus* (MT663219) was 16,345 bp in length and biased toward A and T, with an 80.8% A + T content. The mitogenome of *E. vegetus* contains 13 protein-coding genes (PCGs), 22 tRNA genes, and 2 rRNA genes. Seven tRNAs (*trnC*, *trnY*, *trnF*, *trnH*, *trnP*, *trnV*, *trnL1*), two rRNAs, and four PCGs (*nad1*, *nad4*, *nad4L* and *nad5*), were located on the N-strand, while the J-strand encoded the remaining. Compared with the ancestral gene arrangement of insects (Boore 1999), *trnI* translocated to upstream of *nad2*, and *trnM* and *trnQ* swapped positions. Most PCGs initiated by ATN codons and all PCGs ended with TAA stop codons. There were five gene overlaps among *trnW*-*trnC* (8 bp), *atp8*-*atp6* (7 bp), *atp6*-*cox3* (1 bp), *nad6*-*cob* (1 bp), *trnS*-*nad1* (2 bp). A total of 1251 bp of intergenic spacer sequences were found in 20 locations and varied in size from 1 to 680 bp, with the longest located between *trnQ* and *trnI*.

Ten unsaturated amino acid sequences (*nad3*, *atp8,* and *nad4L* were excluded) of 45 Hymenoptera were aligned by TranslatorX (Abascal et al. 2010) and concatenated with SequenceMatrix v1.7.8 (Vaidya et al. 2011). The phylogenetic trees were constructed using Bayesian inference (BI) with PhyloBayes (Lartillot et al. 2009) under the MtArtCAT model in CIPRES (Miller et al. 2010) (Figure 1). It revealed Blennocampinae represented by *E. vegetus* is a sister group of Fenusinae which was represented by *Birmella discoidalisa* (MF197548) in Tenthredinidae. All related files have been uploaded to figshare (https://figshare.com/account/home#/projects/83588).

**Figure 1.**
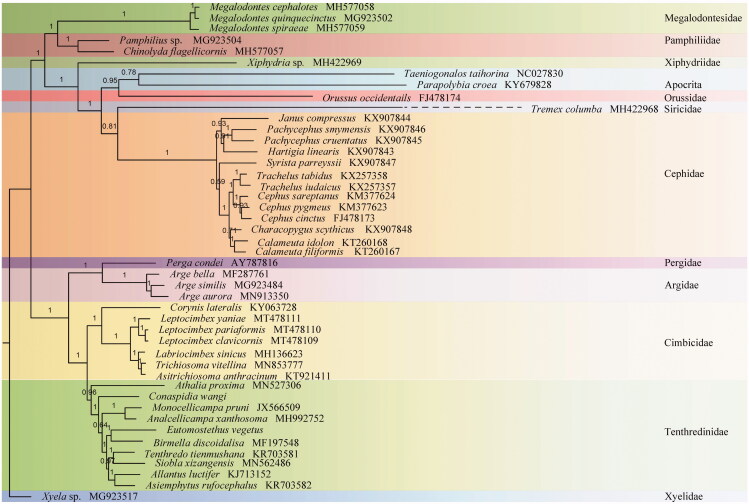
Phylobayes tree based on the combination of ten unsaturated amino acids. The numbers above each node correspond to the posterior probabilities.
